# Is work-family conflict a pathway between job strain components and binge eating? A cross-sectional analysis from the ELSA-Brasil study

**DOI:** 10.1186/s40337-022-00540-x

**Published:** 2022-02-05

**Authors:** Leidjaira Lopes Juvanhol, Aline Silva-Costa, Lucia Rotenberg, Arlinda B. Moreno, Enirtes Caetano Prates Melo, Itamar S. Santos, Maria Angélica Antunes Nunes, Susanna Toivanen, Dóra Chor, Rosane Härter Griep

**Affiliations:** 1 Department of Nutrition and Health, Federal University of Viçosa (UFV), Viçosa, MG Brazil; 2 Federal University of Triangulo Mineiro (UFTM), Uberaba, MG Brazil; 3 Laboratory of Health and Environment Education, Oswaldo Cruz Institute, Rio de Janeiro, Brazil; 4 National School of Public Health, Oswaldo Cruz Foundation (Fiocruz), Rio de Janeiro, RJ Brazil; 5 University of São Paulo (USP), São Paulo, SP Brazil; 6 Federal University of Rio Grande do Sul (UFRGS), Rio Grande do Sul, Brazil; 7 Mälardalen University, Västerås, Sweden

**Keywords:** Eating disorder, Eating behavior, Occupational stress, Job stress, Psychological stress, Obesity

## Abstract

**Background:**

Job strain has been reported as a trigger for binge eating, yet the underlying mechanisms have been unclear. The aim of this study was to evaluate whether work-family conflict is a pathway in the association between job strain and binge eating, considering the possible effect-modifying influence of body mass index (BMI).

**Methods:**

This cross-sectional analysis included 12,084 active civil servants from the multicenter Brazilian Longitudinal Study of Adult Health (ELSA-Brasil). Job strain was assessed using the Demand-Control-Support Questionnaire. Work-family conflict was considered as a latent variable comprising three items. Binge eating was defined as eating a large amount of food in less than 2 h at least twice a week in the last six months with a sense of lack of control over what and how much was eaten. Structural equation modelling was used to test the role of work-family conflict in the association between job strain and binge eating, stratifying for BMI.

**Results:**

For individuals of normal weight, positive associations were found between skill discretion and binge eating (standardized coefficient **[**SC] = 0.209, 95%CI = 0.022–0.396), and between psychological job demands and work-family conflict (SC = 0.571, 95%CI = 0.520–0.622), but no statistically significant indirect effect was found. In overweight individuals, psychological job demands, skill discretion, and work-family conflict were positively associated with binge eating (SC = 0.099, 95%CI = 0.005–0.193; SC = 0.175, 95%CI = 0.062–0.288; and SC = 0.141, 95%CI = 0.077–0.206, respectively). Also, work-family conflict was observed to be a pathway on the associations of psychological job demands and decision authority with binge eating (SC = 0.084, 95%CI = 0.045–0.122; and SC =  − 0.008, 95%CI =  − 0.015– − 0.001, respectively).

**Conclusions:**

Work-family conflict partly explains effects of high levels of psychological job demands and low levels of decision authority on binge eating among overweight individuals. Moreover, skill discretion is positively associated with binge eating, regardless of BMI category.

## Plain English summary

Recent studies have found work-family conflict (i.e., incompatible work and family demands) to link between work issues and physical and mental health. Accordingly, this study investigated whether the relationship between job strain and binge eating is explained by work-family conflict, by body mass index (BMI), in a large sample of Brazilian civil servants. Overall, this study demonstrated that, among overweight individuals, excessive job demands and low decision authority (over what to do at work and how) contribute to binge eating by increasing work-family conflict. Also, excessive skill discretion at work, including opportunities to acquire and use specific job skills, is related to binge eating, regardless of BMI, which deserves further investigation. In conclusion, the results indicate that work-family conflict is a potential mechanism through which job strain can affect eating behavior among overweight individuals.

## Background

A binge eating episode is defined as eating a large amount of food within a limited period of time, with a sense of lack of control over what and how much is being consumed [1]. Recurrent binge eating episodes are the core feature of two important eating disorders, bulimia nervosa, and binge eating disorder, for which lifetime prevalence estimates are 1.0% and 1.9%, respectively, according to the World Mental Health Survey Initiative [2], and the trend is increasing [3]. Binge eating is often associated with other physical and mental comorbidities, including obesity, diabetes mellitus, anxiety, and depressive symptoms [4, 5]. It is thus a major public health concern, especially among obese individuals, which displayed significantly higher prevalence of this behavior [4, 6, 7].

Psychological stress [8], including job strain [9], has been reported as a trigger for binge eating. Job strain results from the combination of high psychological demands and low control over the work process [10]. Job psychological demands refers to the load and pace of activities and the difficulty of performing them, as well as conflicting demands. Job control, meanwhile, comprises two dimensions: skill discretion (opportunities to be creative, use intellectual competences and develop new skills) and decision authority (opportunities to take decisions about work) [11].

Another important work-related issue is work-family conflict, i.e., “a form of inter-role conflict in which the role pressures from work and family domains are mutually incompatible in some respect” [12]. Although studies of work-family conflict and binge eating were not identified, work-family conflict has been associated with health outcomes, such as poor self-rated health [13], mental illness [14], and unhealthy eating [15]. Recently, authors have suggested that work-family conflict is a partial mediator in the relationship between work-related stress and mental health [16–18].

Accordingly, it was hypothesized that: i) work-family conflict is a pathway in the association between job strain components and binge eating, and ii) body mass index (BMI) acts as an effect modifier in this relationship. Therefore, based on a structural equation modelling approach, this study evaluated whether work-family conflict is a pathway in the association between job strain and binge eating, considering the possible effect-modifying influence of BMI.

## Methods

This is a cross-sectional analysis of baseline data (2008–2010) from 12,084 active civil servants in the Brazilian Longitudinal Study of Adult Health (ELSA-Brasil), aged 35–74 years, sampled from five universities and one research institution in Brazil. The ELSA-Brasil cohort study examined the development of chronic diseases, especially diabetes and cardiovascular disease [19]. As the course of these diseases is long, individuals aged under 35 years were not included in the study, so as to avoid excessively long follow-up. The study included information obtained during comprehensive interviews conducted by trained personnel and was performed under stringent quality assurance and control procedures [20, 21].

Job strain components were assessed using the Brazilian version of the Swedish Demand‐Control‐Support Questionnaire [22], a 17-item short version of the Job Content Questionnaire, with responses given on a four‐point Likert‐scale. The three-dimensional scale estimated: (i) psychological job demands (comprising five items: work quickly, work intensely, work effort, available time, and conflicting demands), (ii) skill discretion (three items: learning new things, skill level, and scope to take initiative), and (iii) decision authority (two items: how to do the work and what to do at work). As suggested by Hökerberg et al. [23, 24], the best-fit model was achieved by removing the social support at work dimension (six items) and the item repetitive work from the skill discretion component, in addition to inserting error correlation between the working fast and working intensely items (psychological demands). Each component was used as a continuous variable, with higher scores indicating higher levels in each dimension.

Work-family conflict, a latent variable, included three items [25, 26]: the first, time-based work-family conflict (WFC1), was assessed by the question: *Do demands (requirements or requests) from work keep you from spending the amount of time you would like with your family?*; the second question focused on strain-based work-family conflict (WFC2): *Do demands (requirements or requests) from work make it difficult to fulfill domestic responsibilities, such as caring for the house and children?*; and the third question assessed the simultaneous effects of both work and family on lack of time for leisure and self-care (WFC3): *Do demands (requirements or requests) from your family and work keep you from spending the amount of time you would like on your own care and leisure activities?* Answers were given on a five‐point scale (never to almost never, rarely, sometimes, often, and very often) and were grouped into three levels: “never” (never to rarely, reference category); “sometimes”; and “often” (often and very often) [13, 27].

Binge eating was assessed by the question “*Some people, at certain times, eat a large amount of food at once in a short time (up to two hours). They feel they have lost control of their eating, that is, they cannot avoid starting to eat, and after starting, cannot stop. During the past six months, how often did you eat this way?”* proposed by Freitas et al. [6]. The response categories were: *never, less than once a week, once a week,* and *twice a week or more*. Binge eating was defined as the occurrence of binge eating episodes twice a week or more in the prior six months, in accordance with the Structured Clinical Interview for DSM-IV – SCID-I/P [28].

**Fig. 1:**
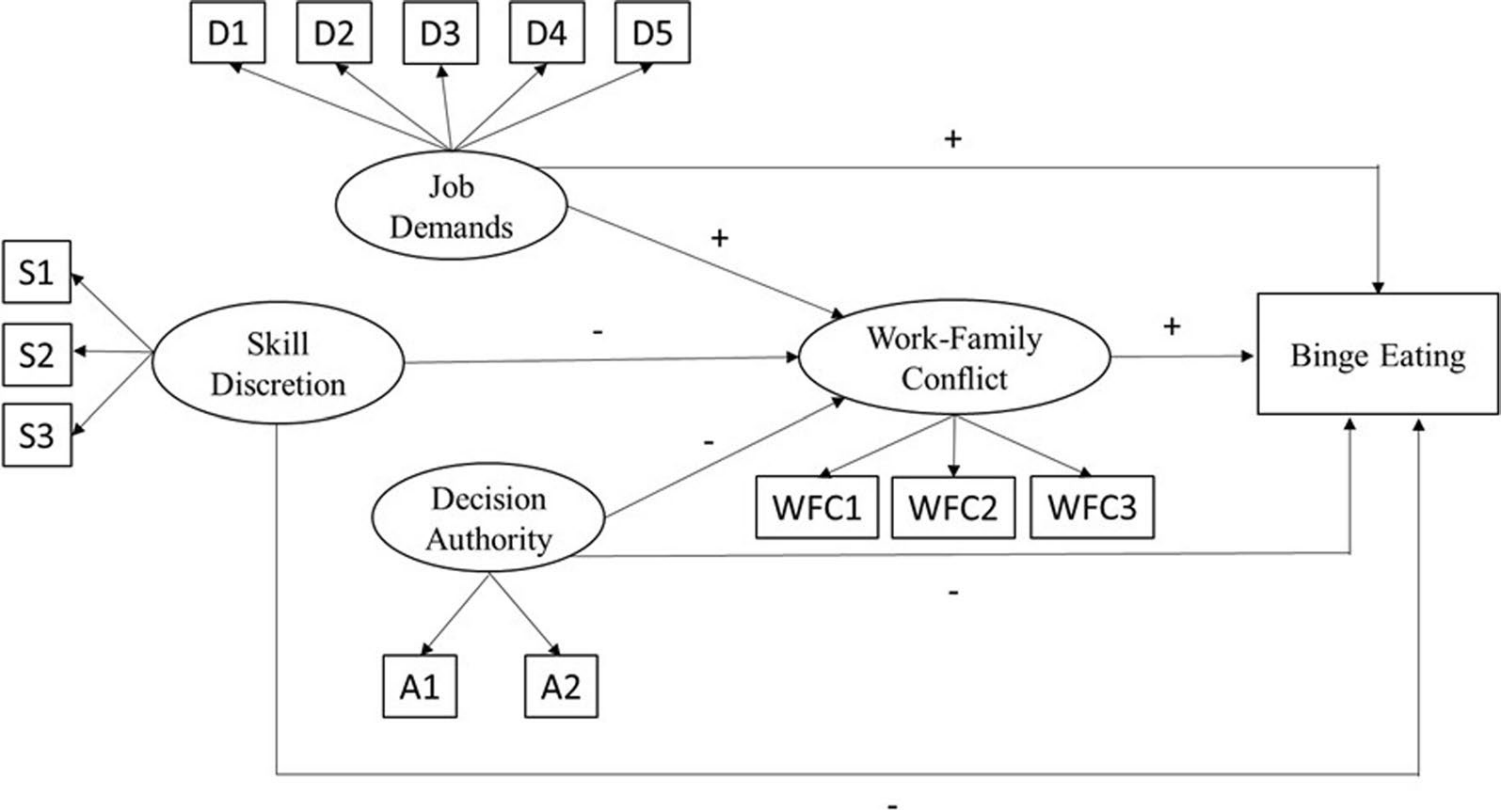
Theoretical model tested using structural equation modeling. *A1* deciding how to do the work; *A2* deciding what to do at work; *D1* having to work very fast; *D2* having to work very intensively; *D3* work demanding too much effort; *D4* having enough time to do everything; *D5* work involving conflicting demands; *S1* learning new things through work; *S2* work demanding a high level of expertise; *S3* job requiring initiative; *WFC1* time-based interference of work with family; *WFC2* strain-based interference of work with family; *WFC3* lacking time for personal care and leisure due to family and work demands

Structural equation modeling (SEM) was used to test whether work-family conflict is a pathway in the association between job strain and binge eating, according to the theoretical model proposed (Fig. 1). The weighted least squares mean and variance adjusted (WLSMV) estimator was used for parameter estimation, as recommended for categorical variables. The standardized coefficients (SCs) were estimated with their respective 95% confidence intervals (95%CI). Statistical significance was determined by the 95%CI, with an effect considered significant when zero was not included within the CI. Model fit was evaluated using the Tucker-Lewis index (TLI) and the comparative fit index (CFI), in which values above 0.90 indicate good model fit [29], and the root mean square error of approximation (RMSEA), with values < 0.06 indicating good fit [30]. Factor loadings for the latent variables were considered acceptable when higher than 0.30 and statistically significant (*p* < 0.05) [29].

Considering previous results from the study population—which found interaction with BMI, but not with sex, in the relationship between job strain and binge eating [9]—multiple-group analysis was performed to test potential differences between normal weight (BMI < 25 kg/m^2^) and overweight (BMI ≥ 25 kg/m^2^) individuals. Testing for cross-group invariance was by chi-square difference test to compare two nested models: (i) a baseline model where no constraints were specified; and (ii) a constraint model, in which parameter estimates were assumed to be equal across the two groups [31].

Also included, as potential confounders, were age (continuous), education (less than high school, complete high school, college, and postgraduate), and sex. The software used was R, version 2.15, and Mplus, version 7.4. The DIFFTEST option was used for multiple-group analysis in Mplus, as described by Muthén and Muthén [32].

All study procedures were carried out in accordance with the ethical standards of the Brazilian National Research Ethics Committee. Informed consent was obtained from all participants included in the study.

## Results

The mean age of the study population was 49.2 years (standard deviation = 7.3 years), 52.2% were women and 36.7% had postgraduate education. The prevalence of binge eating was 6.9%. After stratifying participants by BMI, prevalence of binge eating was 9.4% and 2.7%, respectively, among overweight and normal weight individuals.

The factor loadings for the latent variables (i.e., job strain components and work-family conflict) were high for most items in both groups (normal weight and overweight) and all factor loads were positive and statistically significant. The item conflicting demands in psychological job demands showed the lowest of all loadings of the job strain components (normal weight = 0.381 and overweight = 0.436). In relation to work-family conflict, the item WFC3 (lack of time for personal care and leisure) showed the lowest factor loading (normal weight = 0.691 and overweight = 0.709) and the item WFC1 (time-based interference of work with family) showed the highest factor loading (normal weight = 0.893 and overweight = 0.873) (Table 1).

**Table 1:** Structural equation model^a^ for the association between job strain components, work-family conflict and binge eating

	Standardized coefficients (95%CI)	
	Normal weight (n = 4567)	Overweight (n = 7517)
	Latent loadings	
**Psychological job demands**		
D1: working fast	0.581 (0.551–0.611)	0.587 (0.565–0.610)
D2: working intensely	0.709 (0.683–0.734)	0.683 (0.663–0.703)
D3: work effort	0.729 (0.705–0.753)	0.725 (0.705–0.744)
D4: available time	0.585 (0.553–0.617)	0.576 (0.550–0.603)
D5: conflicting demands	0.381 (0.350–0.412)	0.436 (0.413–0.460)
**Skill discretion**^b^		
S1: learning new things	0.539 (0.505–0.574)	0.503 (0.475–0.531)
S2: skill level	0.746 (0.718–0.774)	0.747 (0.724–0.770)
S3: taking initiative	0.724 (0.694–0.753)	0.745 (0.722–0.768)
**Decision authority**		
A1: how to do the work	0.750 (0.720–0.780)	0.757 (0.733–0.782)
A2: what to do at work	0.811 (0.779–0.842)	0.779 (0.754–0.803)
**WFC**		
WFC1: time-based interference of work with family	0.893 (0.875–0.910)	0.873 (0.858–0.887)
WFC2: strain-based interference of work with family	0.861 (0.842–0.879)	0.843 (0.827–0.858)
WFC3: lack of time for personal care and leisure	0.691 (0.666–0.715)	0.709 (0.690–0.728)
**Factor correlations**		
Psychological job demands ↔ Skill discretion	− 0.440 (− 0.487– − 0.394)	− 0.503 (− 0.538– − 0.467)
Psychological job demands ↔ Decision authority	0.018 (− 0.024–0.060)	0.054 (0.021–0.088)
Decision authority ↔ Skill discretion	0.439 (0.393–0.486)	0.386 (0.348–0.423)
Error measurement correlation D1 ↔ D2^b^	0.373 (0.335–0.412)	0.413 (0.385–0.442)

	Direct effects	
Psychological job demands → binge eating	0.012 (− 0.133–0.157)	0.099 (0.005–0.193)
Skill discretion → binge eating	0.209 (0.022–0.396)	0.175 (0.062–0.288)
Decision authority → binge eating	0.047 (− 0.077–0.171)	− 0.033 (− 0.105–0.039)
WFC → binge eating	0.111 (− 0.002–0.223)	0.141 (0.077–0.206)
Psychological job demands → WFC	0.571 (0.520–0.622)	0.592 (0.547–0.637)
Skill discretion → WFC	0.028 (− 0.060–0.116)	0.077 (0.004–0.149)
Decision authority → WFC	− 0.026 (− 0.085–0.033)	− 0.057 (− 0.103– − 0.010)
**Indirect effects**		
Psychological job demands → WFC → binge eating	0.063 (− 0.001–0.127)	0.084 (0.045–0.122)
Skill discretion → WFC → binge eating	0.003 (− 0.007–0.013)	0.011 (0.000–0.022)
Decision authority → WFC → binge eating	− 0.003 (− 0.010–0.004)	− 0.008 (− 0.015– − 0.001)
**Total effects (direct + indirect)**		
Psychological job demands → binge eating	0.075 (− 0.041–0.191)	0.183 (0.106–0.259)
Skill discretion → binge eating	0.212 (0.027–0.398)	0.186 (0.073–0.298)
Decision authority → binge eating	0.044 (− 0.060–0.168)	− 0.041 (− 0.112–0.031)
**Model fit**		
RMSEA (90% CI)	0.045 (0.043–0.046)	
CFI	0.958	
TLI	0.948	

*CFI* comparative fit index; *RMSEA* root mean square error of approximation; *TLI* Tucker-Lewis index; *WFC* work-family conflict; *A1* deciding how to do the work; *A2* deciding what to do at work; *D1* having to work very fast; *D2* having to work very intensively; *D3* work demanding too much effort; *D4* having enough time to do everything; *D5* work involving conflicting demands; *S1* learning new things through work; *S2* work demanding a high level of expertise; *S3* job requiring initiative; *WFC1* time-based interference of work with family; *WFC2* strain-based interference of work with family; *WFC3* lacking time for personal care and leisure due to family and work demands

^a^The model was also adjusted by age, education, and sex

^b^In line with previous findings, the item repetitive work (skill discretion) was excluded and an error correlation between D1 and D2 (psychological job demands) was included

For normal weight individuals, positive associations were found between skill discretion and binge eating (SC = 0.209, 95%CI = 0.022–0.396), and between psychological job demands and work-family conflict (SC = 0.571, 95%CI = 0.520–0.622), but no statistically significant indirect effect was found in this group. Among overweight individuals, psychological job demands, skill discretion, and work-family conflict were positively associated with binge eating (SC = 0.099, 95%CI = 0.005–0.193; SC = 0.175, 95%CI = 0.062–0.288; and SC = 0.141, 95%CI = 0.077–0.206, respectively). Also, work-family conflict was observed to be a pathway in the association of psychological job demands and decision authority with binge eating (SC = 0.084, 95%CI = 0.045–0.122; and SC =  − 0.008, 95%CI =  − 0.015– − 0.001, respectively). Lastly, considering the total effect, psychological job demands and skill discretion were associated with binge eating (SC = 0.183, 95%CI = 0.106–0.259; and SC = 0.186, 95%CI = 0.073–0.298, respectively) among overweight individuals. Among normal weight individuals, skill discretion was associated with binge eating (SC = 0.212, 95%CI = 0.027–0.398) (Table 1).

The unconstrained model provided adequate fit to the data (RMSEA = 0.045, 90% CI = 0.043–0.046; CFI = 0.958; and TLI = 0.948) (Table 1). Constraining the model parameters to be equal across the normal weight and overweight groups resulted in a statistically significant chi-square difference test (chi-square value = 43.292; *df* = 21; *p* = 0.003), thus rejecting the null hypothesis that model parameters are the same by BMI.

## Discussion

To our knowledge, this is the first study to investigate work-family conflict as a pathway in the relationship between job strain components and binge eating. The findings support the hypothesis that high levels of psychological job demands and low levels of decision authority have effects on binge eating that, among overweight individuals, are partly explained by work-family conflict. Also, for normal and overweight groups, the positive direct effects of skill discretion on binge eating deserve attention.

Given the epidemic of metabolic diseases, for which unhealthy diet, sedentarism, and environmental stress are risk factors [33], it is fundamental to understand the mechanisms underlying the etiology of eating disorders. Results from a laboratory study showed that stress increased the average eating rate in obese women with binge eating disorder [34], suggesting that individuals may attempt to cope with stressful situations by overeating, as an escape valve [33]. Previous studies have also shown that job strain is positively associated with eating disorders [9, 35], as well as that favorable situations at work, such as those involving rewards (e.g., financial remuneration, esteem, and career opportunities) are negatively associated with overeating [36]. Although the role of occupational stress in binge eating is not well understood, this relation can be explained as a feedback mechanism in which exposure to heavy psychological job demands and lack of control leads to increased levels of the adrenalin and cortisol that regulate eating behaviors and choices [37]. In addition, this eating behavior may result from a specific stress situation relating to an imbalance between work and family demands. In line with the findings of this study, work-family conflict has a significant effect on eating [38]. Work-family conflict is generally associated with multiple detrimental demands, which evoke negative psychological responses [39].

Work-family conflict is a potential source of psychosocial stress and is recognized, in a more complex conceptual framework, as a putative consequence of stress at work [16]. This is proposed because high psychological demands and low control at work may contribute to an absence of boundaries between professional and home life [40]. According to the literature [12], conflicts occur when individuals’ participation in work interferes with their participation in the family. Therefore, job characteristics that exhaust workers undermine the work-family balance, whereas job characteristics that help workers to cope with work demands can prevent the adverse effects of work-family conflict [16, 41]. From this perspective, some studies have found work-family conflict to link between work issues and physical and mental health [16, 42, 43]. Extending our previous findings on the association between job strain and binge eating [9], this study adds the knowledge that work-family conflict may be a pathway in this relationship.

This study found job strain and binge eating to be more strongly associated among overweight than normal weight individuals, and the indirect effects through work-family conflict were observed only among the former. It has been suggested that job strain affects normal weight and overweight individuals differently: it has been seen to lead to greater weight gain among the latter and lower weight gain, or even weight loss, among the former [44, 45]. One possible explanation is the differential influence of stress on eating behavior, which may lead to under- or overeating and, in this regard, obese individuals would tend to eat more in response to stress than lean individuals [46]. Job stress has been found to be more strongly associated with over-eating among men with higher BMI while, among those with lower BMI, associations were weak or null [36]. Our results thus support the hypothesis that job strain has a heterogeneous effect on eating behaviors, by BMI. The mechanism by which this differential effect operates remains unknown, but it is well known that obesity and disordered eating are closely related traits [47]. Overweight individuals are more likely to be involved in binge eating episodes, because of more intense body dissatisfaction and weight suppression [48], which may favor a more pronounced response to job strain and work-family conflict among them. Furthermore, evidence indicates that BMI-related genes are also related to binge eating susceptibility [49, 50].

The other main finding of this study was a positive direct association between skill discretion and binge eating in both normal-weight and overweight individuals, contrary to the effects suggested by the demand-control model [51]. Emerging evidence has revealed different and even opposite effects of skill discretion and decision authority on health outcomes [52, 53], where they act as independent constructs. It has thus been suggested that the two subscales of job control be analyzed separately [23, 54], as in this study. The skill discretion scale is used to assess opportunities to be creative, use intellectual competences, and learn new things [11]. However, it has been pointed out that some items of this scale are more indicative of job complexity than job control [55], and work contexts with high levels of skill discretion are seen as characterized by high levels of intellectual demands [56]. It has thus been argued that skill discretion may be perceived as an additional demand rather than as a protective factor, because learning new things and exercising creativity require that workers continuously update and mobilize energy, especially in highly demanding occupations [57]. In line with this, a study of male Japanese factory workers showed that jobs requiring higher levels of concentration and of knowledge and skill (termed the “qualitative workload”) were positively correlated with overeating behaviors, such as eating rhythm abnormalities, feeling of satiety, and motivation for eating [58]. However, given that the ELSA-Brasil population is composed of civil servants with higher levels of formal education than other workers, and whose work involves high levels of psychological job demands and job control [59], the findings of this study require further confirmation in other job settings.

As the aim here was to investigate the effects of work—i.e., job strain components—on the balance between domestic life and health, this study did not include family interference with work in the latent variable “work-family conflict”. The extent to which family life interferes with work is related to disruptive demands that evoke different emotional responses from those triggered by job stressors. Although some authors have suggested that work-family conflict is a source of stress, this study produced consistent significant results in a conceptual framework where work-family conflict is a consequence of job strain components. Nonetheless, given its cross-sectional design, it was not possible to determine causal pathways among job strain, work-family conflict, and binge eating. Longitudinal studies are needed to understand better how modifications in any one of these constructs may affect the others. Also, the data were insufficient to determine clinical diagnoses of bulimia nervosa and binge eating disorder. Accordingly, despite evaluating binge eating, which is a behavior present in these disorders, they could not be identified in this study. To overcome this potential limitation, a more conservative cutoff was used for binge eating frequency (two or more episodes per week), so as to consider only clinically significant cases.

## Conclusions

This study suggests that work-family conflict is a significant pathway in the relationship between high psychological job demands and low levels of decision authority and binge eating among overweight Brazilian civil servants. Moreover, it found that skill discretion has a direct, positive effect on binge eating, regardless of BMI category, which deserves further investigation. It has theoretical and practical implications in that it has built on prior research to further explore work-family conflict as a contributory mechanism to explain the relationships between job characteristics and workers’ health, particularly as regards eating behavior.

This novel study reinforces the argument that job characteristics should be included as a priority in the future workers’ health agenda. Furthermore, it suggests that more attention should be paid to establishing family-supportive workplace initiatives to prevent and attenuate the impact of binge eating and associated disorders. Regarding clinical applicability, our findings demonstrate that interventions to reduce stress at work and in family life, such as cognitive behavioral, coping and mindfulness strategies, may have therapeutic effect on binge eating, especially among overweight individuals.

## Data Availability

The datasets used and analysed during the current study are available upon request to the Study’s Steering Committee, through an appointed representative, Dr. Rosane Härter Griep (rohgriep@gmail.com). The ELSA-Brasil study, while open to any researcher, has a policy of requiring that all proposals of investigations pass through the studýs publications committee.

## Abbreviations

BMI: Body mass index
CFI: Comparative fit index
CI: Confidence interval
ELSA-Brasil: Brazilian Longitudinal Study of Adult Health
RMSEA: Root mean square error of approximation
SEM: Structural equation modeling
SC: Standardized coefficient
TLI: Tucker-Lewis index
WLSMV: Weighted least squares mean and variance adjusted
